# A scoping review of the impact of COVID-19 pandemic on surgical practice

**DOI:** 10.1016/j.amsu.2020.07.003

**Published:** 2020-07-09

**Authors:** Amjad Soltany, Mohammed Hamouda, Ansam Ghzawi, Ahmed Sharaqi, Ahmed Negida, Shaimaa Soliman, Amira Yasmine Benmelouka

**Affiliations:** a Department of Plastic and Reconstructive Surgery, Al Mouwasat University Hospital, Faculty of Medicine, Damascus University, Damascus, Syrian Arab Republic; b Faculty of Medicine, Alexandria University, Alexandria, Egypt; c Faculty of Medicine, Yarmouk University, Irbid, Jordan; d Faculty of Medicine, Mansoura University, Dakahlia, Egypt; e School of Pharmacy and Biomedical Sciences, University of Portsmouth, Portsmouth, UK; f UK and Faculty of Medicine and Zagazig University Hospitals, Zagazig University, Sharkia, Egypt; g Public Health and Community Medicine Department, Faculty of Medicine, Menoufia University, Menoufia, Egypt; h Faculty of Medicine, University of Algiers, Algiers, Algeria

**Keywords:** Coronavirus, Surgery, Emergency, World health organization, Telemedicine

## Abstract

**Background:**

The current COVID-19 pandemic has challenged the infrastructure of the healthcare systems. To cope with the pandemic, substantial changes were introduced to surgical practice and education all over the world.

**Methods:**

A scoping search in PubMed and Google Scholar was done using the search terms: “*Coronavirus,” “COVID-19”, “SARS-CoV-2”, “nCoV-2019”, and “surgery.”* They were either searched individually or in combination. All relevant articles of any study design (published within December 15, 2019, till the mid of June 2020), were included and narratively discussed in this review.

**Results:**

Sixty-six articles were reviewed in this article. Through these articles, we provide guidance and recommendations on the preoperative preparation and safety precautions, intraoperative precautions, postoperative precautions, postoperative complications (related to COVID-19), surgical scheduling, emergency surgeries, elective surgeries, cancer surgery, psychological impact on surgical teams, and surgical training during the COVID-19 pandemic.

**Conclusion:**

COVID-19 pandemic has affected nearly all aspects of surgical procedures, scheduling, and staffing. Special precautions were taken before, during, or after surgeries. New treatment and teaching modalities emerged in response to the pandemic. Psychological support and training platforms are necessary for the surgical team.

## Highlights

• Strict surgical protective measures should be taken to avoid viral transmission.
• Decision to postpone elective surgeries is a multidisciplinary one.
• Mental and psychological support is essential for surgeons during the pandemic.
• The pandemic profoundly impacts surgical practice and training.
• Guidelines to improve surgical practice during the pandemic are rapidly evolving.

## 1 Introduction

Coronavirus disease (COVID-19) is a global pandemic affecting over 3 million people and has vastly impacted healthcare systems worldwide. The World Health Organization (WHO) officially recognized this public health issue as an international emergency on March 11, 2020 [1]. The surgical field has been influenced as a result of the massive redirection of medical attention and priority towards caring for the COVID-19 infected patients. The economic demands of this global issue are increasing, and they are being exacerbated by the needs of surgical procedures [2]. Also, surgeons are now facing the challenge of maintaining their care and working to reduce the nosocomial spread of the virus.

On March 12, 2020, the United States of America (USA), Center for Disease Control and Prevention (CDC) recommended canceling all the elective surgeries at Santa Clara County, California [3]. Further, many general guidelines were released to set the criteria of case classification in order to define elective surgeries that can be postponed and urgent procedures that need a rapid intervention [4,5]. Surgeons should decide on the indications basing on a case-by-case approach. Moreover, in order to avoid person-to-person contact, experts suggested taking benefit from the recent technologies in making remote consultations, patients’ allocation and triage, educating junior surgeons, and knowledge sharing [6].

The timing of cancer surgery is critical to limit tumor progression, prevent or delay metastases, and improve patient survival. Therefore, cancer surgeries, which might be life-saving, are of particular concern during the current pandemic. The decision on delaying the resection should be discussed by the responsible surgical team that should, in this case, take into consideration the risk of nosocomial contamination with SARS-CoV-2, and the risk of tumor spread and metastasis [7].

This narrative review provides a global view of the impact of the COVID-19 pandemic on the surgical field. We summarized the safety measures required in the different operative and postoperative phases. We also highlighted the impact of COVID-19 on elective and cancer surgery scheduling and the evolvement of the process of taking surgical decisions. Finally, we discussed the impact of the pandemic on the surgical staff mental health and education.

## 2 Methods

We conducted a scoping review of published literature on the impact of COVID-19 pandemic on surgical practice and training. We also aimed to review the evolvement of the principals of decision-making regarding the possibility of postponing or performing different surgical procedures. The search included all relevant articles from December 2019 till the mid of June 2020.

### 2.1 Literature search

A computer literature search of PubMed and Google Scholar was done using the following keywords: “*Coronavirus”, “COVID-19”, “pandemic”, “surgical”, “surgery”, “elective surgery”, “emergency surgery”, “surgical training”, “surgical preparation”, “surgeons”, and “surgical residents”*. They were either used individually or in combination.

### 2.2 Scope and criteria

We included all relevant articles about the impact of COVID-19 on any of the following surgical domains [1]: preoperative preparation and safety precautions [2], intraoperative precautions [3], postoperative precautions [4], postoperative complications (related to COVID-19) [5], surgical scheduling [6], emergency surgeries [7], elective surgeries [8], cancer surgeries [9], psychological impact on surgical teams, and [10] surgical training. We included published articles that are available in the English language, of any study design as well as the WHO related reports, and the guidelines for surgical practice released by credited institutions or professional associations. Studies were classified according to their scope, their findings, and recommendations. They were tabulated and discussed narratively.

**Fig. 1:**
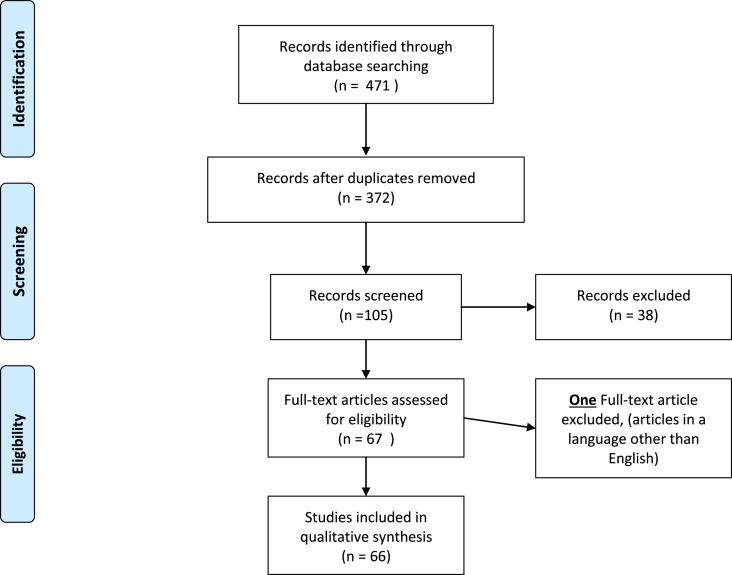
PRISMA 2009 flow diagram explains the selection process

In this review, we followed the checklist of the “Preferred Reporting Items for Systematic Reviews and Meta-Analyses Protocols (PRISMA)” [8]. The selection process is explained by the PRISMA flow diagram (Fig. 1).

## 3 Results

The literature search identified 471 articles. Of them, 66 articles and reports were eligible for inclusion in this scoping review (related to surgical practice during the COVID-19 pandemic). The summary of the included articles is shown in Table 1.

**Table 1:** shows a summary of the articles included in this scoping review

Scope	Study ID	Place or professional society	Article type or study design	Key points
Preoperative preparation & safety precautions	Combira R. et al, 2020	European Society of Trauma and Emergency Surgery (ESTES)	Guidelines/Recommendations	- Recommendations for perioperative surgical preparation for the COVID 19 pandemic. - Extensive safety precautions to be followed by the surgical team for safe, adequate, and efficient surgical practice. - Full protective attire is recommended. - The most experienced surgeons are encouraged to be performing the operations themselves for shorter operative time and low risk of complications.
	Saadi RA et al, 2020	USA	Guidelines/Recommendations	- N95 Masks, eye protection, and PAPR usage are mandatory when dealing with infected ENT patients.
	Givi B et al, 2020	USA	Review article and Recommendations	- Safety guidelines for Head and Neck service. PPE is crucial.
	Pichi B et al. 2020	Italy	Guidelines/Recommendations	- CORONA acronym (C= cover yourself, OR= operating room settings, O= open the trachea, NA= nursing, and airway management), a stepwise approach for tracheostomy management in infected patients.
	Forrester JD et al, 2020	USA	Guidelines/Recommendations	- Training staff for donning and doffing is mandatory. - A tree design algorithm of OR precautions is designed to maximize safety and efficiency.
Intraoperative precautions	Ti LK et al, 2020	Singapore	Letter to the editor	- A runner outside the OR should be available to service the OR. - A minimum of 1 hour between the cases is mandatory
Postoperative precautions	Tan Z et al, 2020	Singapore	Recommendation/Guidelines	- Dedicated OR complexes should be available for COVID-19 patients. - Staff should shower before resuming regular activities. - Patients should be transferred wearing full PPE
	Sica GS et al.	Italy	Debate article	- The authors observed that, since the beginning of the pandemic, the patients have become more compliant with the enhanced recovery program.
	Wong J et al, 2020	Singapore	Review article	- Phone calls can replace post-op visits. - In situ simulations are exercised to train staff members for upcoming stressful events in resuscitation and management of critical cases whilst wearing full PPE. - Consent and Charting are done using touch screens for easier decontamination.
Postoperative complications (related to COVID-19)	Aminian A et al, 2020	Iran	Case series	- Three out of four infected patients died after developing ARDS within two weeks of their surgical procedure
	Fukuhara S et al, 2020	China	Retrospective cohort	- Thirty-four infected patients had undergone elective surgeries. 44.1% required ICU care. The mortality rate was 20.5%. The most common causes of death were ARDS, shock, acute cardiac injury, and arrhythmia.
	COVIDSurg Collaborative 2020	International study	Cohort study	- Perioperative COVID-19 infection is associated with a high rate of pulmonary complications and with a high mortality.
Surgical scheduling	Topf MC et al, 2020	USA	Recommendations/Guidelines	- Providing a framework for prioritization criteria for otolaryngologic surgeries during the COVID-19 pandemic and discussing preoperative clinical strategies for transmission reduction and the role of preoperative COVID-19 testing.
	COVIDSurg Collaborative 2020	International study	A global expert-response study	- The COVID-19 pandemic will lead to the cancellation of an enormous number of interventions.
	Yu GY 2020	China	Recommendations/strategies	- Patients with colorectal cancer should undergo surgery as soon as possible after resuming elective surgeries. - Recommending laparoscopy-assisted radical surgery for colorectal cancer patients
	Stahel PF et al, 2020	USA	Editorial	- Suggesting a decision-making algorithm for risk-stratification of elective surgical procedures and showing that reconciliation between the interpretation of “elective, non-urgent” and patient’s health could be a challenge
	American College of Surgeons, American Society of Anesthesiologists, Association of periOperative Registered Nurses, and American Hospital Association, 2020	USA	Joint statement/ strategies	- Roadmap for resuming elective surgeries after COVID-19 pandemic
	Wiseman SM et al. 2020	Canada	Commentary	- A big number of surgical interventions have been cancelled or delayed in Canada. - It is necessary to implement a surgical wait list and to prepare for its management after the pandemic.
	Zarrintan S et al, 2020	Iran	Correspondence	- In epidemic areas, elective surgeries should be suspended unless the complications of the disease could lead to serious risks
	American college of Surgeons, 202	USA	Guidelines/ Recommendations	- Suggested guidelines for local resumption of elective surgery
Emergency surgeries	Lisi G et al, 2020	Italy	Correspondence	- COVID19 has led to a major reduction in the number of surgeries and surgical services worldwide. - Non-urgent operations have been canceled temporarily and have been given less priority. - Delaying the surgery of colorectal malignancies could lead to serious outcomes.
	Combira R. et al, 2020	European Society of Trauma and Emergency Surgery (ESTES)	Recommendations	- Every acute admission must be evaluated by at least two surgeons (consultants, attendees) to assess the risk of proceeding in comparison to the risk of delay, and to decide the need for alternative interventions. - Limiting the delay of intervention while maintaining the quality must be taken into consideration in decision making.
	Patriti A et al, 2020	Italy	Letter to the editor	- Lockdown has resulted in a reduction in the number of surgeries in Italy by 86%. - Lockdown and cancellation of surgeries have led to tragic consequences in patients with emergent severe surgical symptoms.
	Pryor A et al, 2020	USA	Recommendation	- Despite the emergency due to the pandemic, emergent surgeries must not be delayed. - Cases must be evaluated by a multidisciplinary team to prioritize the patients’ need for surgery.
	Stahel PF ,2020	USA	Editorial	- The authors propose a decision-making algorithm to stratify surgical procedures during the pandemic.
	Topf MC et al, 2020	USA		- The identification of four categories of patients according to the level of urgency 1.urgent: surgery should be done at the time 2.less urgent: postponement of the surgery for more than 30 days is to consider 3.less urgent: the surgery can be postponed for 30–90 days 4. the use of a case-by-case basis is recommended.
	Lisi G et al, 2020	USA	Guidelines	- Blanket policies are not advised for surgical triage. - Evidence and expert opinion from qualified clinicians and administrators should be taken into consideration to perform case triage.
Elective surgeries	Topf MC et al, 2020	USA	Recommendations/Guidelines	- Providing a framework for prioritization criteria for otolaryngologic surgeries during the COVID-19 pandemic and discussing preoperative clinical strategies for a transmission reducing and the role of preoperative COVID-19 testing.
	Yu GY et al , 2020	China	Recommendations/strategies	- Patients with colorectal cancer should undergo surgery as soon as possible after resuming elective surgeries. Recommending laparoscopy-assisted radical surgery for colorectal cancer patients
	Stahel PF et al, 2020	USA	Editorial	- Suggesting a decision-making algorithm for risk-stratification of elective surgical procedures and showing that reconciliation between the interpretation of “elective, non-urgent” and patient’s health could be a challenge
	American College of Surgeons, American Society of Anesthesiologists, Association of periOperative Registered Nurses, and American Hospital Association, 2020	USA	Joint statement/ strategies	- Roadmap for resuming elective surgery after COVID-19 pandemic
	Zarrintan S et al,2020	Iran	Correspondence	- In epidemic areas, elective surgeries should be suspended unless the complications of the disease could lead to serious risks
	American college of Surgeons, 2020	USA	Guidelines/ Recommendations	- Local resumption of elective surgery guidance
Cancer surgery	Gillessen S et al, 2020	Europe	Editorial	- Systemic therapy of uro-oncology cases can be delayed due to difficulty or uncertainty. - Neoadjuvant therapy may be beneficial for uro-oncology patients who cannot undergo surgery or radiotherapy because of the pandemic
	Liang W et al, 2020	China	Comment	- The possibility of postponing adjuvant chemotherapy or elective cancer surgery for stable cancer. - Cancer patients should have more protection - Patients with cancer patients with COVID19 should have intensive treatment and surveillance.
	Mehta V et al, 2020	USA	Observational study	- Cancer patients with COVID-19 are at a high risk of case fatality.
	Kuderer NM et al, 2020	USA	Cohort study	- The mortality among cancer patients who are SARS-COV-2 positive depends on some general risk factors and on risk factors unique to the cancer as well.
	Di Saverio et al, 2020	Italy	Guidance	- Lessons learned from the experience Italian surgeons regarding colorectal surgery during the pandemic. - The selection of patients undergoing proctological procedures and endoscopy should be done with caution. - The use of conservative approaches is advised in managing colorectal emergencies. - The surgical treatment of COVID19 positive patients should be done using high protective measures.
	Sharma et al. 2020	India	Review Article	- Chemotherapy or surgery may be associated with higher rate of COVID-19 infection.
	van Harten MC et al, 2014	Germany	Retrospective cohort	- Delaying the treatment of head and neck squamous cell carcinoma up to 90 days does not affect the survival.
	Samson P et al, 2015	USA	Retrospective cohort	- The delayed resection of non-small lung cell carcinoma (stage I) is associated with higher comorbidity scores.
	Grotenhuis BA et al, 2010	Northlands	Cohort Study	- The delay of diagnosing and treating esophageal cancer is associated with poor outcomes.
	Van Harten MC et al, 2015	Northlands	Retrospective cohort	- Delaying the treatment of head and neck squamous cell carcinoma is associated with poor outcomes.
	Robinson KM et al, 2012	Denmark	Survey study	- Treatment delay may affect the quality of life and the survival of patients with ovarian or endometrial cancer.
	Bartlett DL et al, 2020	USA	Editorial	- Considerations of managing cancer surgeries during the pandemic.
	De Felice F et al, 2020	Italy	Correspondence	- Strategies for treating advanced rectal cancer during the pandemic include: Short-course radiotherapy followed by surgery after 5 to 13 weeks. Standard long-course treatment should be maintained for the T4 stage.
	Ueda M et al,2020	USA	Special feature	- The article describes the importance of the organizational structure, the preparation, the agility, and the application of shared vision to continue providing cancer treatment.
	Ciavattini A et al, 2020	Italy	Special article	- Recommendations about the evaluation of patients with cervical lesions according to cytology. - The use of technology to share colposcopic images with reference centers is recommended. - The use of the lowest possible energy in electrosurgical instruments is recommended.
	Stensland KD et al, 2020	USA	Editorial	- The article describes the average length of stay of urologic cancer patients according to the current medical evidence.
	Ficarra V et al, 2020	Italy	Short communication	- Recommendations about the general urologic practice during the pandemic.
	Campi R et al, 2020	Italy	Retrospective cohort	- The possibility of postponing two-thirds of elective uro-oncologic surgeries or changing the treatment to another modality.
	Pellino G et al, 2020	Italy	Viewpoint	- The effect of COVID19 pandemic on colorectal cancer treatment in Italy.
	Çakmak GK 2020	Turkey	Editorial	- Many factors have been taken in consideration in managing breast surgery during COVID-19 era in Turkey. - There was a net reduction in breast cancer surgeries in high volume centers in Turkey.
	Sullivan M et al, 2020	Child cancer organizations: SIOP,SIOP-E,COG,SIOP-PODC,IPSO,PROS, ICPCN, St Jude Global, and the WHO	Special report	- Measures and practical advice for managing children with cancer during this pandemic.
	Downs et al, 2020	Australia	Correspondence	- During the pandemic, decisions on cancer surgery are rapidly evolving. - Onco-surgery procedures should be continued when possible and some considerations should be taken in order to assure an optimal care. - The use of hospital recorders and robust database is necessary to follow up the patients whom surgeries have been delayed.
	The Society of Thoracic Surgeons and the American Association for Thoracic Surgery, 2020	USA	Consensus Statement	- Recommendations about thoracic malignancies operations triage. - Alternative treatment strategies are recommended instead of surgical resection
	Fregatti P et al, 2020	Italy	Observational clinical study	- The use of careful selection criteria of patients and preventive measures can help to accomplish safe surgeries for breast cancer.
	Sud et al, 2020	United Kingdom/ the data was from Wuhan	Predictive design analysis	- The delay of cancer surgeries may have an impact on patients’ prognosis.
	Chang et al, 2020	USA	Prospective and retrospective assessment of cancer surgery cases	- A net reduction oncologic surgeries was engendered by the COVID-19 pandemic.
	Cenzato M et al, 2020	Italy	Editorial	- The article describes modalities of the use of online networking in neuro-surgery during the pandemic
	Fakhry N et al, 2020	France	Recommendations	- The definition of the groups of patients with head and neck cancer based on the treatment time scale. - The release of organizational aspects regarding consultations, hospitalizations and surgeries of patients with head and neck cancer.
Psychological impact on surgical teams	Xu J et al, 2020	China	Pre-proof	- Anxiety, Depression, Dream anxiety, and SF-36 quality of life scales among the front-line hospital staff before and after the COVID-19 outbreak and found that all the scores after the outbreak were significantly higher.
	Neto MLR et al, 2020	Brazil	Review article	- Nurses, doctors, healthcare workers, and other medical professionals are at a higher risk of getting infected than the general public. - Psychological suffering and other mental health symptoms are challenging health care professionals.
Surgical training	Tomlinson SB et al, 2020	USA/India	Editorial	- The annual meeting of the American Association of Neurological Surgeons (AANS) canceled. - Exams administered by many universities and institutes like the American Board of Neurological Surgery was postponed. - Institutional suspensions of critical research activities. - Visitors to online 3D neurosurgical atlas increased by more than 20%
	Chick RC et al, 2020	USA	Perspectives	- The Facebook group titled “ABSITE Daily” members increased from 27 to 237with more than 120 daily views. - Online platforms offering video teleconferencing, lectures, case conferences, journal clubs, and audible podcasting are the main methods in this new era of telemedicine.
	Kogan M et al, 2020	USA	Standard Review	- Recommendation of social distancing and virtual education. - Expanding usage of smart technology for distance learning.
	Porpiglia F et al, 2020	Europe	comment	- Nonessential elective surgeries and procedures were postponed and limited only to non-deferrable procedures. - Surgical opportunities for residents were reduced.
	Amparore D et al, 2020	Italy	Review article	- Residents’ training was critically affected. - Strategies aiming to increase the use of telemedicine, smart learning programs and telementoring of surgical procedures are warranted to address this challenge.

## 4 Discussion

### 4.1 Preoperative preparation and safety precautions during COVID-19 pandemic

Many articles in the literature have emphasized on the importance of having a separate operating room (OR) complex designated to serve COVID-19 infected patients going for a surgical procedure [[9], [10], [11], [12], [13]]. These operating theaters should be set at negative pressure interiorly, unlike the standard conventional positive pressure rooms, which aids in preventing the spread of SARS-COV-2 outside the OR. The designated OR complex may be away from the main OR complex in order to limit the occurrence of any cross-contamination among patients. The OR doors should be shut at all times as far as the surgery is taking place, and the number of individuals entering and exiting the OR should be strictly limited [11].

All equipment that will be potentially used for any procedure must be inside the OR prior to the patient's entry [9,11,13]. Spare instruments and devices inside the OR are beneficial in decreasing the likelihood of any staff member moving in or out of the OR [11]. Disposable equipment has been used as an alternative to reusable ones [9].

Regarding the staff attire, appropriate Personal Protective Equipment (PPE) was always a standard precaution taken during whenever medical personnel was dealing with a COVID-19 patient. Several papers heavily emphasized this practice [[9], [10], [11], [12], [13], [14]]. The variations in the literature were very minimal, as most of the hospitals already require a disposable cap, gown, gloves, N95 mask, and a face-shield for dealing with the infected patients in intensive care units (ICU) or the preoperative isolation ward. PPE donning was performed at a specially designated area in close vicinity to the OR [13]. An article by Pichi et al. [15] described a “buddy check” where two individuals check each other to ensure both were wearing full PPE. A mandatory PPE donning and doffing training was also mentioned in a study by Forrester et al. [16]. Wong et al. also mentioned an in situ simulation training to test the preparedness of the healthcare staff to manage COVID-19 patients while in full PPE and Powered Air-Purifying Respirator (PAPR) to address any unapparent problem that may arise in a real event later on [13].

The patients should be transferred directly without having to wait in the preoperative holding area [9]. Charting and consent should be done electronically using touch screen devices for easier decontamination and lower risk of infection spread through pens, papers, etc. [13]. Having a separate elevator designated to carrying COVID-19 patients was mentioned as well [9]. Patients should be transferred while wearing a gown, gloves, N95 mask, and eye protection. All additional staff should be out of the OR during intubation, and doors should be closed for 10 min post-intubation [11].

### 4.2 Intraoperative precautions during COVID-19 pandemic

All staff members in the OR should be wearing full PPE, double gloves, and an extra surgical mask over the N95, followed by Powered Air-Purifying Respirator for maximum protection (PAPR) is recommended [9]. Two studies mentioned a runner being available by phone to serve the OR either by entering directly or by the use of an anteroom. The materials that are being transferred to the OR (e.g., instruments) or out of it (e.g., frozen sections) placed on a trolley in the anteroom for the runner or the staff in the OR to retrieve hence limiting direct contact with each other [9,12]. The most experienced surgeons are advised to perform those surgeries on infected patients to ensure operations done in the timeliest manner and reduce the possibility of intraoperative complications [9]. Electrocautery and laser use should be limited as much as possible in order to reduce the generated fumes. The smoke produced during the usage of electrocautery should be eliminated by special evacuators [9].

### 4.3 Postoperative precautions during COVID-19 pandemic

Extubation and recovery should be made in the OR, and the route to the ICU or isolation ward should be cleared by security. A minimum of 1 h between cases is advised for thorough decontamination of the operating rooms [12]. The healthcare staff members involved are encouraged to change the scrubs after each case [9] and to shower before resuming normal activities [11]. When possible, Postoperative visits should be replaced with phone calls [13]. In order to reduce the in-hospital stay, enhanced recovery programs have also been applied in many centers. A recent report demonstrated that patients could cope easily with these new protocols since they are aware of the infectious risk [17].

### 4.4 Postoperative COVID-19 related complications

The literature regarding COVID-19-related postoperative complications is insignificant. Postoperative acute respiratory distress syndrome (ARDS), shock, arrhythmias, acute cardiac injury, or even death of COVID-19 patients were recorded in some studies [18,19].

COVID-19-positive patients undergoing surgery may be the subject of major postoperative complications, even if they do not have any symptoms of respiratory infection [18,19]. This is why more precautions are required in performing surgical procedures for COVID-19 patients in order to avoid the severe problems that can, consequently, consume healthcare resources.

The international cohort study by the COVIDSurg Collaborative reported that the 30-day mortality was 23.8% among COVID-19 patients who underwent surgery. The pulmonary complication occurred in more than half of the studied patients (51.2%), among whom the mortality was 38.0%. They observed higher mortality rates among males, patients aged 70 years or more, patients graded by the American Society of Anesthesiologists (ASA) from 3 to 5, malignant disease surgeries, emergency surgeries or major surgeries [20]. The results of this COVIDSurg study imply that the threshold for surgery should be higher for COVID-19 patients since they are at greater risk of more complications and mortality.

### 4.5 Surgical scheduling during the COVID-19 pandemic

It is clear -without any doubt-that emergency and trauma surgical patients should be managed during this pandemic without any delay, yet reconciliation between the interpretation of “elective, non-urgent” and patient's health could be a challenge. For this, we are discussing (I) COVID -19 and elective surgeries and (II) COVID-19 and surgical emergencies.

#### (I) Elective surgeries during the COVID-Pandemic

In response to the current pandemic of coronavirus disease (COVID-19), several official institutions have canceled or postponed elective and non-urgent surgical procedures. This reduction has its advantages as increasing capacity for COVID-19 patients in general wards and intensive care units, reducing the risk of cross-infection between COVID-19 patients and other patients, supporting emergency care, and preserving PPE [21].

A Bayesian β-regression model included 71 countries, estimated the surgical cancellation rates during the 12-week lockdown to be 23⋅4–77⋅1% for cancer surgeries, 71⋅2–87⋅4% for non-cancer surgeries, and 17⋅4–37⋅8% for obstetrics surgeries. The same study expected a median of 45 weeks for the world to recover from this backlog of operations if the baseline surgical rates increased by 20% [22].

Surgeries for benign tumors -confirmed with pathological reports-can be postponed, but the whole surgical team should decide surgeries for malignant tumors. Reconstructive and plastic surgeries could be delayed. Unless life-threatening, most orthopedic, urologic, and neurosurgical operations can also be suspended. Palliative surgical management of gastrointestinal obstructions should not be delayed. Concerning vascular surgery conditions like catheter dialysis placement in renal failure patients, ruptured arterial aneurysms, severe deep venous thromboembolism (DVT) associated with phlegmasia should be managed emergently or urgently; whereas, other conditions like most of the venous and lymphatic procedures, aortoiliac occlusive disease, and peripheral arterial disease could be delayed [7].

It may be essential for each surgical specialty to have clear algorithms and frameworks to guide the surgeon's decision-making for proper surgical care. For example, the Division of Head and Neck Surgery in the Department of Otolaryngology at Stanford University has stratified head and neck cases by urgency into four major categories: urgent-proceed surgically, less urgent – consider postponing >30 days, less urgent – consider postponing 30–90 days, and case-by-case basis [21].

On the other side, the entity of elective surgery delaying could have a more negative impact on the patient's health than mortality and morbidity caused by COVID-19. A publication by the Naval Medical University highlighted the major risks of postponing surgeries for colorectal cancer during the COVID-19 period [23,24].

**Fig. 2:**
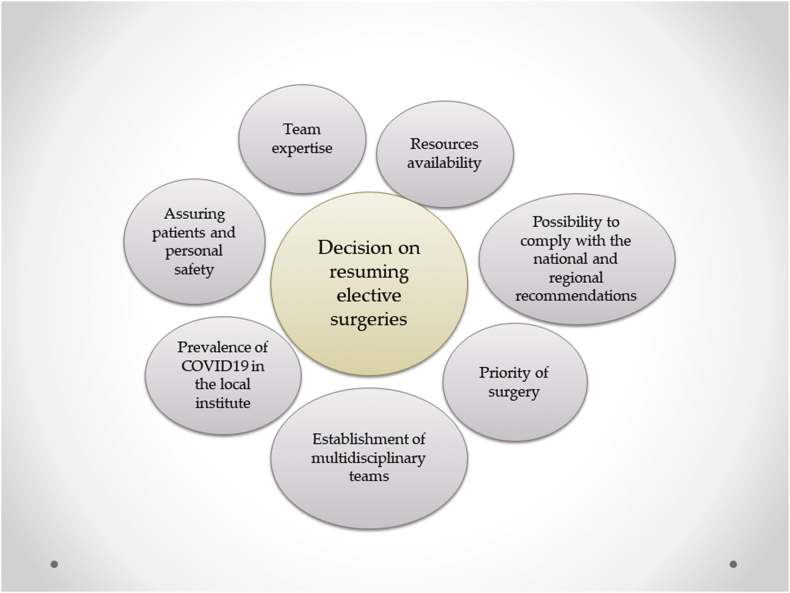
General considerations for resuming elective surgeries.

The American College of Surgeons, the American Society of Anesthesiologists, the Association of perioperative Registered Nurses, and the American Hospital Association have announced a joint statement (April 17, 2020) to guide the roadmap for resuming elective surgeries after COVID-19 pandemic [25]. Besides, the American College of Surgeons has published guidance for the resumption of elective surgeries (April 17, 2020) [26]. The first decisions on postponing all the elective surgeries have led to the generation of waiting lists that included thousands of patients [27]. Consequently, professionals are now focusing on generating protocols to resume elective procedures. These protocols are taking in consideration all the possible scenarios for each hospital. For instance, some teams have generated specific scores to help in achieving this process. In front of all these evolving recommendations, all the surgical teams are now asked to take many considerations before deciding on the resumption of elective surgeries. These considerations include the personal expertise, the risk of infection to both staff and patients, the national and regional guidelines, and the availability of the resources (Fig. 2) [25,26].

#### (II) Emergency surgeries during the COVID-19

The definition of emergency surgery has always been a relevant issue even before the era of COVID-19. Despite all the efforts of the global institutions to establish a comprehensive definition, there is always a place for a case-dependent subjective evaluation by surgeons. To overcome this amid the pandemic, several institutions issued recommendations about decision making and definitive criteria to decide the severity of the presented case. Every acute admission must be evaluated by at least two surgeons (consultants, attendants) to assess the risk difference between proceeding and delaying, and to weigh the role of alternative interventions. A summary of some of the most common surgical emergencies was listed in Table 2 [9,21,24,28,29].

**Table 2:** List of different types of surgical emergencies.

Case Example	Urgency	Indication
Emergent	Less than 1 h	Life-threating emergencies
		Acute exsanguination/hemorrhagic shock
		Trauma level 1 activations
		Acute vascular injury or occlusion
		Aortic dissection
		Emergency C-section
		Acute compartment syndrome
		Necrotizing fasciitis
		Peritonitis
		Bowel obstruction/perforation
Urgent	More than 24 h	Appendicitis/cholecystitis
		Septic arthritis
		Open fractures
		Bleeding pelvic fractures
		Femur shaft fractures & hip fractures
		Acute nerve injuries/spinal cord injuries
		Surgical infections

As mentioned previously, most of the surgical admissions have been limited to include only urgent and emergent life-threatening conditions. As a result, COVID-19 has resulted in a significant reduction in the number of surgical admissions worldwide [24]. Due to the lack of sufficient evidence regarding the impact of the pandemic on surgical emergency and the lack of clear global definitive criteria for the later in the era of COVID-19, the random cancellation of most surgeries has led to unpleasant consequences [30].

Most of the global and regional institutes started to stress on this issue in their guidelines and recommendations. The European Society of Trauma and Emergency Surgery (ESTES) new recommendations have stated that “care should be taken to limit delay of interventions and to maintain quality of interventions’ [9].

The question has been raised here whether to request COVID-19 testing for emergent surgery cases or not. The general rule is to test all surgery patients upon admission; however, surgeons should not delay the time of initiation waiting for test results. In fact, during this pandemic, all patients should be considered COVID-19 positive, and precautions must be adequately considered in all cases [28,31].

### 4.6 Cancer surgeries during the COVID-19 pandemic

The surgical management of cancer patients during the period of COVID-19 is imposing critical challenges to the medical personnel. On the one hand, the increasing demands of handling the pandemic of coronavirus affect the capacities of managing the surgical plans of patients with malignant diseases [32]. On the other hand, cancer patients are more susceptible to develop severe infections leading to an invasive procedure and poor outcomes [[33], [34], [35]]. Also, cancer surgeries expose the patients to the risks of perioperative complications, which necessitate intensive postoperative care, longer hospitalizations, and increasing the probability of infection [18,36,37]. However, data from a recent study showed that the exposure of this category of patients to any surgery is not associated with death [35].

**Fig. 3:**
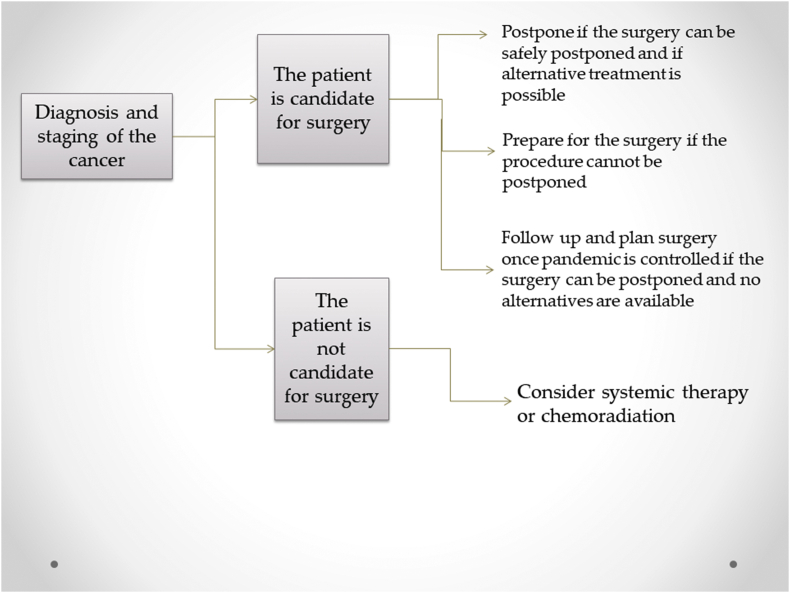
The general approach of managing newly diagnosed cancer patients.

In some types of cancer, deferred surgical care may be considered [36,38]. Nevertheless, in some cases, the delay of the excision may have a great impact on an individual's prognosis and quality of life [[39], [40], [41]]. The need to wait for the surgery may also increase anxiety rates among this specific category of patients [42]. Therefore, the decision of delaying or operating will be challenging and would require a multidisciplinary approach in order to consider all the aspects of each case, including the type and stage of cancer, the age, the physical status, the psychological issues, and the availability of alternative treatments. Fig. 3 summarizes the global approach that is being used in many centers to manage the treatment of newly diagnosed cases with solid tumors. Recommendations to address this issue consider many factors as access to resources, patients' aspects, and disease stage [30,[43], [44], [45], [46], [47], [48], [49], [50], [51], [52]]. Nevertheless, the knowledge and the decisions regarding cancer surgery are rapidly evolving every day. The Society of Surgical Oncology (SSO), as well as other societies and centers, is still appealing for a case-by-case approach in final decision making [43,53].

In general, programmed interventions for an early-stage disease can be deferred in most cases. Furthermore, the application of alternative tools such as neoadjuvant therapies, when possible, has been encouraged by many scientific societies [43,54]. A study with observational data showed that careful selection of patients for elective breast cancer surgery, along with the rigorous application of in-hospital protective measures, could be associated with encouraging outcomes [55].

In all cases, patients should be provided with transparent information about the risks of delaying their surgical care [54]. However, it was recently admitted that postponing incident cancers surgeries for three to six months is expected to mitigate 19–43% of the survival years that can be gained by a rate of hospitalization equivalent to the rate of admissions for COVID-19 patients [56]. Therefore, some authors are now recommending not to delay the surgery for all patients and to ensure coordination among health authorities and professionals to ensure adequate resource allocation [56,57].

In terms of cancer emergencies, current data on the impact of timing is still scarce, but as of now, current recommendations advise to delay all the emergent procedures as far as possible [36]. COVIDSurg Collaborative has conducted a multicenter prospective cohort study to assess the outcomes of cancer surgery in COVID-19 patients. However, the study data have not been published yet. This study will provide guidance for surgeons on the outcomes and risks of cancer surgery in COVID-19 patients.

Moreover, in order to reduce the risk of contamination among this population, there were some interesting initiatives such as the neuro-oncological hub, which is used to achieve conference calls and to share neuro-radiological imaging data. This platform is now used to categorize patients according to the disease severity in order to decide on the rapidity and the modality of surgical care [58]. Another hub structure was established for colorectal cancer cases [18]. In addition to these measures, some experts recommended the postponement of post-cancer surgery face-to-face consultations and replacing them with teleconsultation [59]. To sum up, many considerations should be taken in the management of cancer surgery during this era, and enhanced research work is urgently needed in order to understand the consequences of surgical delays and to establish evidence-based approaches to ensure optimal care for patients who require surgery.

### 4.7 The psychological impact of COVID-19 on the surgical staff

Since WHO has raised the assessment of the global spread and risk of COVID-19 virus to “very high” and after 231,000 deaths had occurred in more than 200 countries and after the sudden increase in confirmed cases and the possibility of transmission via many routes with the vulnerability of surgeons in getting an infection during the long periods of contact with patients, tremendous stress and anxiety states affected the mental health of the surgical medical staff [60].

The surgical medical staff of Baoshan Branch of Shanghai Shuguang Hospital compared the anxiety, depression, dream anxiety, and SF-36 Quality of life scales among the front-line hospital staff before and after COVID-19 outbreak and found that all the scores after the outbreak were significantly higher (P < 0.001) [60].

It is not applicable to test all the patients in emergency rooms for COVID-19. This puts surgeons and anesthesiologists in danger of being infected during intubation or other invasive procedures. This represents a huge psychological burden on medical staff and on their families, as well [61]. Therefore, it is essential to pay attention to the mental health of medical personnel, which may negatively affect their critical medical decisions. Surgical medical staff is also one of the forces to resist the pandemic, so adequate rest and early psychological intervention measures should be provided [62].

### 4.8 Surgical training and the role of telemedicine during the COVID-19 pandemic

Over 3 million cases of COVID-19 have been documented across a total of 210 countries at the time of writing this article. This pandemic is challenging the infrastructure of medical education in light of the recommendations of social distancing and virtual education by CDC. Although medical education is considered a core mission of academic medical centers, an era of expanding usage of smart technology for distance learning, in which social distancing seems the most effective measure, has begun [[62], [63], [64]].

In the USA, all non-essential elective surgeries and procedures are postponed and limited only to non-deferrable procedures. This is reducing surgical opportunities for residents in some departments (e.g., dermatology, urology, …etc.) and increasing others (e.g., trauma, intensive care units). In Europe, urology residents do not have the opportunity to carry out clinical activities nor to be guided as the senior and expert physicians are engaged in emergency management to reduce the operative time and the risk of complications. Most facilities are minimizing participants in any operation to essential personnel only [65,66].

Clinical discussions and the department's meetings were canceled to avoid gathering. The safety of laparoscopic and robotic surgical procedures started to be questioned. Since World War II, the Annual Meeting of American Association of Neurological Surgeons (AANS) was never canceled but for this first time. Exams administered by many universities and institutes like the American Board of Neurological Surgery were postponed. In addition, institutional suspensions of critical research activities are progressing [65].

There is a dilemma with maintaining surgical resident education while providing a safe environment for residents, educators, and patients. Online platforms offering video teleconferencing, lectures, case conferences, journal clubs, and audible podcasting are the main methods in this new era of Telemedicine. Visitors to online 3D neurosurgical atlas increased by more than 20% since the COVID-19 outbreak, 45% of visitors between 25 and 34 years old, mostly medical students and residents whose learning is affected by the global pandemic. The Facebook group titled “ABSITE Daily” members increased from 27 to 237 with more than 120 daily views. This group provides daily practice questions and surgical-related topics virtual discussion about preparing residents for the American Board of Surgery In-Training Examination (ABSITE). In Italy, doctors checked electronic records of patients and picked up cases whose appointments should not be delayed. Consultations were done via phone mainly or face-to-face visits in some cases. Forty-five percent of scheduled consultations were canceled. History taking, formulating the management plan, discussing the case, and counseling can be done via video conference, so the lecturer can see who is currently attending and respond immediately to a question, which gives the feel of an in-person meeting from a safe distance [63].

## 5 Conclusion

Delivery of surgery during this critical period of the COVID-19 pandemic is imposing plenty of challenges on surgeons and surgical practice. It affected nearly all aspects of surgical procedures, scheduling, and staffing. Special precautions to prevent the viral spread and decrease postoperative complications were therefore taken before, during, or after surgeries. New treatment and teaching approaches emerged in response to the pandemic. However, research efforts are still needed to understand the impact of the pandemic and to make evidence-based decisions on performing the procedures. Last, psychological support and training platforms are necessary to enhance the performance of the surgical team.

## Ethical approval

Our manuscript is a narrative review, so there are no patients.

## Sources of funding

No funding was received for this work.

## Author contribution

All of the authors contributed in all the phases of preparing this scoping review.

## Trial registry number

Our manuscript is a narrative review, so there are no patients.

## Guarantor

Ahmed Negida, MBBCh.

## Provenance and peer review

Not commissioned, externally peer reviewed.

## Declaration of competing interest

No conflicts of interest to declare.
